# Correlation Between Thyroid Nodule Size and Risk of Thyroid Cancer: A Retrospective Cohort Study at a Tertiary Care Center

**DOI:** 10.3390/diagnostics16101505

**Published:** 2026-05-15

**Authors:** Osama Zeidan, Talal Sarhan, Zeid Alkhairi, Omar Abusedera, Qaswar Sudani, Hasan Kadhem, Jenan Obaid, Alexandra E. Butler

**Affiliations:** 1 School of Medicine, Royal College of Surgeons in Ireland-Bahrain, Busaiteen 15503, Bahrain; 21202365@rcsi.com (O.Z.); 22200268@rcsi.com (T.S.); 22201494@rcsi.com (Z.A.); 22200354@rcsi.com (Q.S.); 22200093@rcsi.com (H.K.); jenan.e.obaid@gmail.com (J.O.); abutler@rcsi-mub.com (A.E.B.); 2 King Hamad University Hospital, Muharraq P.O. Box 24343, Bahrain; 3 Research Department, Royal College of Surgeons in Ireland, Medical University of Bahrain (RCSI-MUB), Manama P.O. Box 15503, Bahrain

**Keywords:** thyroid nodule, malignancy risk, nodule size, retrospective study, risk stratification, ultrasound

## Abstract

**Background:** Thyroid nodules are common, yet only a small proportion are malignant. The independent role of nodule size in malignancy risk remains debated, particularly after adjustment for clinical, biochemical, and sonographic features. **Methods:** A retrospective cohort study was conducted on adult patients with thyroid nodules evaluated between 2018 and 2025 at a tertiary care center. Clinical, laboratory, ultrasound, cytology, and histopathology data were extracted. Thyroid-stimulating hormone (TSH), free thyroxine (free T4), and sonographic characteristics were analyzed. Univariable and multivariable logistic regression were performed. Missing ultrasound data were addressed using multiple imputation (m = 20), with pooled estimates derived using Rubin’s rules. The final multivariable model included 446 patients. **Results:** A total of 446 patients were included, of whom 91 (20.4%) had thyroid malignancy. Malignant nodules were significantly larger than benign nodules (2.30 [1.80] cm vs. 1.80 [1.13] cm; *p* = 0.015). In univariable analysis, TSH, free T4, and multiple ultrasound features were associated with malignancy. In multivariable analysis, nodule size remained the strongest independent predictor of malignancy (adjusted odds ratio [aOR] 1.51; *p* < 0.001). Hypoechogenicity (aOR 2.07; *p* = 0.020) and microcalcifications (aOR 1.86; *p* = 0.047) also remained independently significant, whereas thyroid function parameters were not associated with malignancy after adjustment. **Conclusions:** Thyroid nodule size is the strongest independent predictor of malignancy, with select ultrasound features retaining additional predictive value. These findings support incorporating nodule size more prominently into thyroid cancer risk stratification while maintaining key sonographic features.

## 1. Introduction

Thyroid nodules are among the most prevalent clinical findings in modern practice, with ultrasound-detected prevalence ranging from 19% to 68% depending on population and imaging technique [1,2]. Despite this high prevalence, the incidence of thyroid cancer among nodules remains relatively low at approximately 4–7%, creating a diagnostic challenge: how to reliably identify the malignant subset while minimizing unnecessary interventions in the benign majority [3]. The increasing prevalence of thyroid nodules may also be viewed within the broader context of the global obesity epidemic and associated cardiometabolic disorders, which have been linked to subclinical thyroid dysfunction and increased thyroid nodule occurrence [4,5]. Emerging evidence further suggests that even among euthyroid individuals, body composition parameters may correlate significantly with thyroid function-related markers [6].

The clinical assessment of thyroid nodules has evolved over the past two decades. Contemporary approaches emphasize risk stratification using ultrasonographic characteristics coupled with selective cytology. The American Thyroid Association (ATA) and American College of Radiology Thyroid Imaging Reporting and Data System (ACR TI-RADS) guidelines highlight features such as microcalcifications, irregular margins, marked hypoechogenicity, and taller-than-wide morphology as indicators of increased malignancy risk [4,5]. However, these systems demonstrate variable predictive performance across different populations and healthcare settings.

Nodule size has long been recognized as a potential risk factor. International guidelines recommend varying intervention thresholds: the ATA guideline suggests consideration of biopsy for nodules >1 cm with suspicious ultrasound features [7,8]. In contrast, some centers adopt higher size thresholds for operative management and routinely recommend diagnostic surgery or thyroidectomy for nodules measuring at least 4 cm, even when cytology is benign, based on concerns about increased malignancy risk and false-negative fine-needle aspiration rates [9,10,11]. Some observational studies report no significant correlation between nodule size alone and malignancy risk [12], while others demonstrate that a nodule size of ≥1 cm exhibits greater aggressiveness and increased metastatic potential, especially in younger patients [13].

While thyroid nodules are common globally, in the Gulf region, the epidemiology of thyroid cancer demonstrates unique patterns, including a notably high incidence among females [14,15]. Most international risk stratification guidelines, however, are derived from North American or European cohorts [7,8]. While some local studies have examined ultrasonographic features in Bahraini patients, validation of established international risk stratification paradigms in this specific population remains essential for optimizing clinical care and resource allocation [16].

This study aimed to quantify the prevalence of thyroid cancer across different nodule sizes at the study center, assess the independent contribution of nodule size to malignancy risk, compare the predictive value of nodule size with qualitative ultrasound features, and identify demographic and clinical factors influencing the risk of malignancy.

## 2. Materials and Methods

### 2.1. Study Setting and Population

This was a retrospective cohort study conducted at a tertiary-care center. Patient data were extracted from the electronic medical record system for patients evaluated between January 2018 and September 2025. The study protocol was approved by the local institutional ethics committee. Patient confidentiality was strictly maintained throughout the data collection and analysis process, with all identifiers anonymized prior to statistical review. The study inclusion and exclusion criteria are summarized in Table 1.

**Figure 1:**
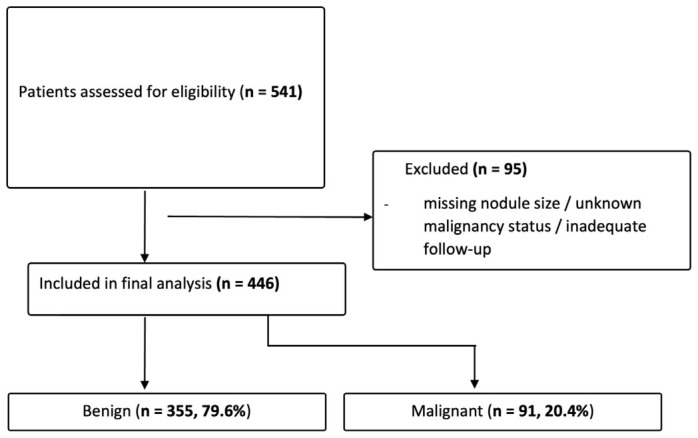
Flowchart of patient selection and classification. A total of 541 patients were initially screened; 95 were excluded based on predefined criteria, yielding a final analytic cohort of 446 patients, of whom 91 (20.4%) had confirmed thyroid malignancy.

Of the patients initially screened (n = 541), 95 were excluded: those with missing or incomplete nodule size measurements, those with unknown or uncertain malignancy status, and those with inadequate follow-up documentation, yielding a final analytic cohort of 446 patients. This information is displayed as a flowchart in Figure 1.

### 2.2. Data Collection and Variables

Data were extracted using a standardized protocol from the database by the study investigators and independently reviewed for accuracy. Different variables were systematically recorded, including the following:

#### 2.2.1. Demographic and Clinical Variables

Demographic and clinical data were extracted from the electronic medical records for all included patients. Collected variables included age at the time of nodule evaluation (years), sex, and nationality. Anthropometric data were recorded as body mass index (BMI, kg/m^2^). Clinical history variables included documented family history of thyroid disease or thyroid cancer, smoking status, and the presence of medical comorbidities, including diabetes mellitus, hypertension, and cardiovascular disease.

#### 2.2.2. Ultrasound Findings

Ultrasound data were extracted from radiology reports at initial evaluation. Nodule size (largest diameter, cm) was recorded as the primary exposure. Additional ultrasound variables included nodule location, multiplicity (solitary vs. multiple), and composition (solid, cystic, or mixed). Qualitative features recorded were echogenicity, microcalcifications, margins, shape (taller-than-wide), and internal vascularity. The ACR TI-RADS category was recorded when available.

#### 2.2.3. Laboratory Data

Thyroid function tests obtained at initial evaluation were recorded, including serum thyroid-stimulating hormone (TSH; mIU/L) and free thyroxine (T4; pmol/L) levels. Patients were classified as hypothyroid, euthyroid, or hyperthyroid based on laboratory values and clinical documentation when available.

Assays were performed using electrochemiluminescence immunoassay (ECLIA) on an analyzer in accordance with standard laboratory protocols at the study institution.

#### 2.2.4. Fine-Needle Aspiration Biopsy and Pathologic Data

FNAB data were collected for nodules undergoing cytologic evaluation. Cytology results were classified according to the Bethesda System (I–VI) and grouped into clinically relevant categories, including benign, atypia of undetermined significance/follicular lesion of undetermined significance (AUS/FLUS), follicular neoplasm, suspicious for malignancy, and malignant.

#### 2.2.5. Surgical Pathology Data

For patients undergoing thyroid surgery, procedure type and final histopathological diagnosis were recorded and categorized as benign or malignant. Malignant tumors were subtyped by histology when available.

#### 2.2.6. Follow-Up and Management Data

Longitudinal clinical records were reviewed to confirm final malignancy status based on histopathology or documented clinical and imaging follow-up. Postoperative management and recurrence status were recorded when available.

### 2.3. Outcomes

The primary outcome was thyroid nodule malignancy (benign vs. malignant). Malignancy was defined by histopathology when available; otherwise, final status was determined using Bethesda cytology with documented longitudinal clinical/imaging follow-up and clinician consensus. Secondary analyses evaluated malignancy risk across predefined size categories and age strata.

### 2.4. Statistical Analysis

Analyses were performed using IBM SPSS Statistics version 26 (IBM Corp., Armonk, NY, USA) with a two-tailed significance level of α = 0.05. Normality was assessed using the Shapiro–Wilk test and histogram inspection. Continuous variables are presented as mean ± standard deviation (SD) or median [interquartile range, IQR] as appropriate and compared using the independent-samples *t*-test or Mann–Whitney U test, respectively. Categorical variables are presented as n (%) and compared using the chi-square test. Univariable logistic regression was used to estimate unadjusted associations with thyroid malignancy, reporting odds ratios (ORs) and 95% confidence intervals (CIs). Variables were selected for multivariable analysis based on *p* < 0.10 in univariable analysis or established clinical relevance. Missing qualitative ultrasound data were identified in 77 of 446 patients (17.3%). Little’s test for missing completely at random (MCAR) was statistically significant (*p* < 0.001), suggesting that the missingness pattern was not completely random. Multiple imputation using fully conditional specification was therefore performed under a missing-at-random (MAR) assumption, generating 20 imputed datasets using a fixed random seed, with pooled estimates obtained using Rubin’s rules. The multivariable logistic regression model included nodule size, TSH, free T4, and age as continuous variables, and sex, multiplicity, hypoechogenicity, microcalcifications, irregular margins, composition, vascularity, and taller-than-wide shape as binary covariates, using the enter method to obtain adjusted odds ratios (aORs). No stepwise selection procedure was applied, and all clinically relevant covariates were retained a priori rather than through data-driven selection. Multivariable analysis was subsequently performed on the full cohort (n = 446) using pooled estimates from the imputed datasets. Multicollinearity was assessed using variance inflation factors (VIFs) across the imputed datasets, with all VIF values below 1.5, indicating no evidence of significant collinearity. As a sensitivity analysis, complete-case logistic regression was performed.

During manuscript preparation, a generative artificial intelligence tool (OpenAI) was used only for language editing and clarity improvement. All scientific content, data analysis, and interpretation were performed by the authors, who take full responsibility for the final manuscript.

## 3. Results

### 3.1. Baseline Characteristics and Demographics

A total of 446 patients with thyroid nodules meeting the inclusion criteria were analyzed. Baseline demographic characteristics are summarized in Table 2A. There were no statistically significant differences in mean age (*p* = 0.340) or mean BMI (*p* = 0.154) between the benign and malignant groups. Clinically, the number of nodules (solitary vs. multiple) was not associated with malignancy risk (*p* = 0.705). Similarly, the presence of comorbidities (*p* = 0.632), family history of thyroid disease (*p* = 0.235), and smoking status (*p* = 0.110) showed no significant association with malignancy.

The overall prevalence of malignancy in the study cohort was 20.4% (91/446). Histological subtyping of the 91 malignant cases revealed: papillary thyroid carcinoma (PTC, all variants) in 79 cases (86.8%), follicular thyroid carcinoma (FTC) in 5 cases (5.5%), Hürthle cell carcinoma in 2 cases (2.2%), medullary thyroid carcinoma in 1 case (1.1%), poorly differentiated thyroid carcinoma in 1 case (1.1%), and 3 cases (3.3%) with mixed or unclassified variant histology (Table 2B). Females comprised 78.5% (n = 350) of the study population.

Of the 446 patients, 353 (79.1%) underwent FNAB cytologic evaluation, and 126 (28.3%) underwent thyroidectomy. Among patients who underwent thyroidectomy, 98 (77.8%) had also undergone a prior FNAB evaluation.

The overall cohort had a mean age of 45.5 ± 13.3 years and a mean BMI of 27.5 ± 5.1 kg/m^2^. Females constituted 78.5% of the study population. Solitary nodules were present in 39.9% of patients, while 60.1% had multinodular disease. Comorbid conditions (diabetes mellitus, hypertension, and cardiovascular disease) were identified in 31.4% of patients; among those with specified comorbidities, hypertension and diabetes mellitus were the most frequently reported conditions. Additionally, 8.8% reported a family history of thyroid disease, and 13.8% had a history of smoking. With no significant difference between benign (116/355, 32.7%) and malignant groups (24/91, 26.4%; *p* ≈ 0.25). Radioiodine therapy was administered post-diagnosis in 29 patients (6.5%); prior external beam radiation exposure was not systematically documented in the retrospective records for the full cohort, which is acknowledged as a study limitation.

### 3.2. Univariate Analysis of Clinical and Sonographic Predictors

Univariate analysis of clinical and sonographic features is presented in Table 3. The median serum TSH level in the malignant group was significantly lower than in the benign group (1.22 [2.57] mIU/L vs. 2.24 [2.20] mIU/L; *p* < 0.001). Free T4 levels also differed significantly between groups (1.45 [0.72] pmol/L vs. 1.29 [0.59] pmol/L; *p* = 0.010).

Nodule size emerged as a strong predictor of malignancy. The median diameter of malignant nodules was significantly larger than that of benign nodules (2.30 [1.80] cm vs. 1.80 [1.13] cm; *p* = 0.015). When stratified by a clinically relevant threshold, nodules >3 cm showed nearly a threefold increase in the odds of malignancy compared to smaller nodules (OR 2.90; 95% CI 1.71–4.93; *p* < 0.001).

Among qualitative sonographic features, microcalcifications (*p* = 0.004), hypoechogenicity (*p* = 0.004), irregular margins (*p* = 0.022), and intranodular vascularity (*p* = 0.013) were significantly associated with malignancy. Taller-than-wide morphology (*p* = 0.102) and solid composition (*p* = 0.608) did not reach statistical significance in this cohort.

The presence of microcalcifications was significantly associated with malignancy (OR 2.19, 95% CI 1.29–3.73; *p* = 0.004), using non-calcified nodules as the reference. Hypoechogenicity was also associated with an increased malignancy risk compared to iso- or hyperechoic nodules (OR 2.16, 95% CI 1.27–3.67; *p* = 0.004).

Irregular margins conferred higher odds of malignancy relative to smooth margins (OR 2.01, 95% CI 1.11–3.66; *p* = 0.022). Additionally, the presence of internal vascularity was significantly associated with malignancy compared with absent vascularity (OR 1.91, 95% CI 1.15–3.18; *p* = 0.013).

### 3.3. Multivariable Logistic Regression Analysis

A multivariable logistic regression model was constructed, adjusting for age, gender, TSH, free T4, and significant sonographic variables, as shown in Table 4. Multivariable logistic regression was performed using multiple imputation (m = 20) on the full cohort (n = 446), with pooled estimates reported using Rubin’s rules.

In this adjusted model, nodule size remained the strongest independent predictor of malignancy. For every 1 cm increase in nodule diameter, the adjusted odds of malignancy increased by 50.7% (aOR 1.51; 95% CI 1.22–1.87; *p* < 0.001). Among qualitative ultrasound features, hypoechogenicity (aOR 2.07; *p* = 0.020) and microcalcifications (aOR 1.86; *p* = 0.047) were independently associated with malignancy. Other sonographic features, including vascularity (*p* = 0.053), irregular margins (*p* = 0.186), and a taller-than-wide shape (*p* = 0.062), did not reach statistical significance. Thyroid function parameters, including TSH (*p* = 0.167) and free T4 (*p* = 0.057), were not independently associated with malignancy in the multivariable model.

### 3.4. Age Subgroup Analysis

**Figure 2:**
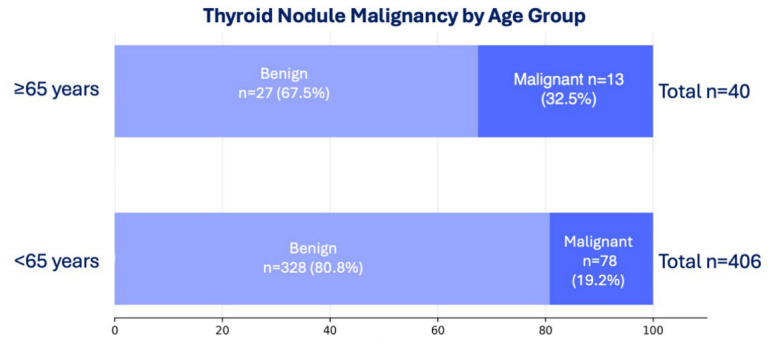
Malignancy risk stratified by age group.

Although mean age did not differ significantly between benign and malignant groups (45.2 vs. 46.7 years; *p* = 0.340), categorical analysis revealed a significant age-related risk disparity (Figure 2). Patients aged ≥ 65 years demonstrated a higher malignancy rate (32.5%; 13/40) compared to those under 65 years (19.2%; 78/406). This difference was statistically significant (*p* = 0.047).

### 3.5. Risk Stratification by Nodule Size Group

**Figure 3:**
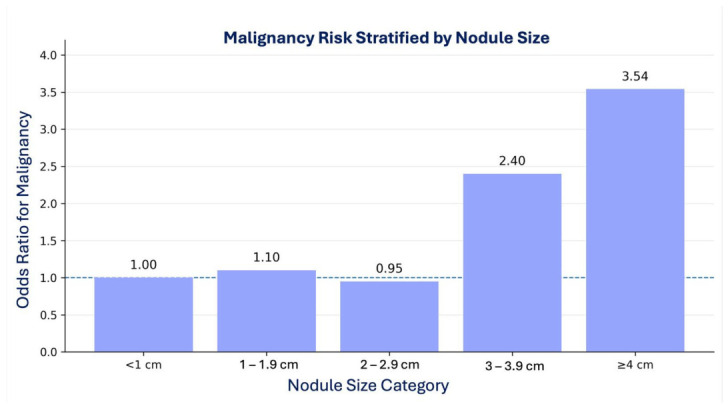
Malignancy risk stratified by nodule size group.

Analysis of malignancy risk by size category revealed a non-linear relationship (Figure 3). The risk remained close to baseline for nodules < 2.0 cm. A distinct inflection point was observed at the 3 cm threshold: nodules in the 3.0–3.9 cm category showed more than double the odds of malignancy (OR 2.40), while those >4.0 cm showed a greater than threefold increase (OR 3.54) relative to the smallest nodules.

The positive predictive value (PPV) for malignancy was 30.6% in the 30–39 mm group and 39.5% in the ≥40 mm group. The negative predictive value (NPV) of a nodule size <30 mm was 84.7%, and the NPV of a nodule size <40 mm was 82.8%, indicating that most nodules below these thresholds were benign.

Among the 197 patients with benign FNAB interpretations (Bethesda Category II), 16 subsequently underwent thyroidectomy; all 16 had benign final histopathological diagnoses, yielding a false-negative rate of 0% in this cohort. In the 30–39 mm size group, 24 patients had benign FNAB results (4 proceeded to surgery, all benign on histopathology; FN rate 0%). In the ≥40 mm group, 13 patients had benign FNAB results (1 proceeded to surgery, confirmed benign; FN rate 0%). These findings indicate high FNAB specificity in this cohort, although the limited number of benign-FNAB patients who proceeded to surgery warrants cautious interpretation of the false-negative rates.

## 4. Discussion

The principal finding of this retrospective cohort study is that nodule size serves as the strongest independent predictor of thyroid malignancy in this study population, while select qualitative ultrasound features also retained independent predictive value in multivariable analysis. Specifically, we identified a non-linear risk progression with a distinct inflection point at 3 cm, where the odds of malignancy nearly triple. Furthermore, while age was not a significant continuous predictor, patients aged ≥65 years represented a distinct high-risk subgroup. These findings challenge the “one-size-fits-all” approach of some international guidelines (ATA, TI-RADS) and suggest that in certain populations, size-based thresholds may need to be lowered to optimize cancer detection [7,8].

Our finding that malignancy risk increases steadily with nodule size aligns with select large series and meta-analyses, though evidence remains mixed across studies. A 2015 meta-analysis of 10,817 nodules showed those measuring 3.0–5.9 cm had 26% higher odds of malignancy versus <3 cm nodules (OR 1.26; 95% CI 1.13–1.39) after adjustment, positioning size as an independent predictor beyond cosmetic concerns [17]. Conflicting data emerge elsewhere: a 2018 study found size mattered mainly in intermediate/low-suspicion US patterns, with ≥3 cm nodules at 40.3% malignancy versus 14.1% for smaller ones, but overall risk trends favored PTC decline and non-PTC rise with size [18]; conversely, a 2019 cohort (503 nodules) saw no size association (OR 0.26 < 3 cm vs. 0.29 ≥ 3 cm, *p* = 0.77), prioritizing cytology class [19], while a 2025 review of 17 studies noted lower malignancy in >4 cm nodules (15% vs. 37%, non-significant RD 0.1) [20]. False-negative FNA rates further complicate interpretation, with multiple published studies reporting higher rates in larger nodules (e.g., 17–30% in ≥3 cm cystic/solid nodules vs. near 0% in smaller solid ones) [21].

Beyond its statistical association, nodule size likely reflects underlying biological and temporal processes that contribute to malignant transformation. Larger nodules may represent lesions with prolonged growth duration, increased cumulative exposure to mutagenic stimuli, or altered tumor microenvironments that favor neoplastic progression. Experimental and clinical studies suggest that increasing tumor volume is associated with hypoxia, angiogenesis, and clonal heterogeneity, factors that may promote aggressive behavior and diagnostic escape on cytology [22,23,24].

Additionally, follicular-patterned lesions, which are more difficult to classify cytologically, tend to present at larger sizes and may contribute disproportionately to malignancy risk in large nodules [25]. This observation aligns with studies demonstrating higher rates of follicular thyroid carcinoma and follicular-variant papillary thyroid carcinoma in nodules exceeding 3–4 cm [26].

**Figure 4:**
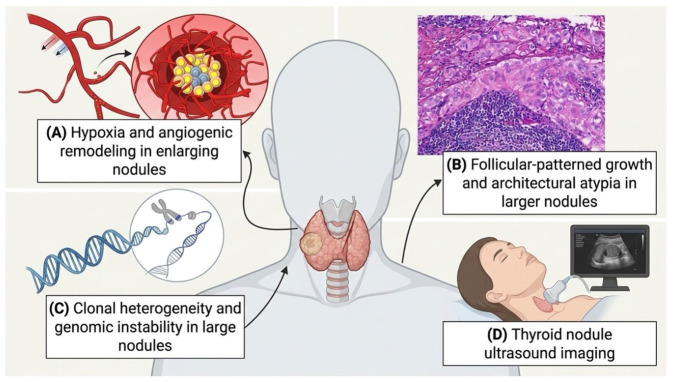
Biological and diagnostic mechanisms linking increasing thyroid nodule size to malignancy risk.

From a diagnostic standpoint, increasing nodule size may reduce FNAB sensitivity due to sampling error, intranodular heterogeneity, and cystic degeneration, reinforcing the observed discordance between cytology and final pathology in large nodules [27]. Our findings therefore support the interpretation of nodule size not merely as a surrogate marker but as a composite indicator integrating biological aggressiveness, diagnostic uncertainty, and temporal evolution. Figure 4 shows a conceptual model illustrating biological and diagnostic mechanisms linking increasing thyroid nodule size to malignancy risk.

Although hypoechogenicity and microcalcifications remained independently associated with malignancy, other ultrasound features lost statistical significance after adjustment. This partially diverges from the weighting of morphology in systems like ACR TI-RADS and ATA, which weigh morphology more heavily than size [4,8]. This divergence may be explained by the “masking effect” in larger nodules; as nodules expand, they often develop cystic degeneration, coarse calcifications, or distorted architecture that can obscure classic malignant features [2]. Similar results were reported in a study that found that in nodules >4 cm, benign and malignant sonographic appearances overlap significantly, reducing the sensitivity of pattern-based recognition [28]. Consequently, relying solely on qualitative features to triage large nodules in this study population could result in missed diagnoses.

Although classical ultrasound features such as microcalcifications, hypoechogenicity, and irregular margins are well-established indicators of thyroid malignancy, their behavior in the multivariable model suggests a mixed pattern of attenuation rather than uniform loss of significance. While some features, including hypoechogenicity and microcalcifications, remained independently associated with malignancy, others lost statistical significance after adjustment for nodule size and correlated imaging variables. This pattern likely reflects shared variance among sonographic features, particularly in larger nodules where morphological overlap increases. This suggests that nodule size may capture part of the diagnostic information conveyed by morphological characteristics, particularly in larger nodules with heterogeneous architecture, where interdependence among sonographic features may reduce the apparent contribution of individual predictors when modeled together. Although TSH and free T4 levels demonstrated significant differences in univariable analysis, neither parameter was independently associated with malignancy after multivariable adjustment.

These findings may also explain discrepancies between our results and prior studies. Many studies reporting strong associations between individual ultrasound features and malignancy rely on univariable analyses or TI-RADS-based frameworks, in which suspicious findings act within weighted composite scoring systems rather than as isolated predictors [29,30]. In contrast, our multivariable logistic regression model simultaneously adjusted for size and correlated sonographic variables, reducing the apparent effect of overlapping predictors. Additional methodological differences, including variation in outcome definitions (cytology versus histopathology) and differences in study populations or referral patterns in tertiary-care settings, may further account for the observed inconsistencies across studies.

The partial attenuation of qualitative ultrasound predictors after adjustment for nodule size has important implications for clinical risk stratification. Current guideline frameworks prioritize morphologic features to determine biopsy and surveillance thresholds; however, our findings suggest that in larger nodules, reliance on sonographic pattern recognition alone may be insufficient. This is consistent with emerging evidence that ultrasound-based risk stratification systems demonstrate reduced diagnostic accuracy in nodules exceeding 3–4 cm [31,32].

Several authors have advocated incorporating size-weighted modifiers into existing risk models, particularly in populations with higher baseline malignancy prevalence or limited cytologic reliability [33]. In this context, our data support a paradigm in which nodule size functions as a key triage variable alongside select sonographic features, prompting earlier biopsy, repeat cytology, or surgical consideration even in the absence of classic high-risk ultrasound features.

Importantly, this approach does not negate the value of established systems such as ACR TI-RADS but rather suggests that size may recalibrate risk estimates when sonographic features are equivocal. Adoption of such an integrated framework could reduce false reassurance and delayed diagnosis, particularly in elderly patients and those managed in tertiary referral settings.

Our finding that patients ≥65 years have a significantly higher malignancy rate (32.5%) stands in contrast to the traditional teaching that thyroid cancer is predominantly a disease of younger adults [34]. Our finding also contrasts with large-scale studies that reported that malignancy risk per nodule is inversely related to age, peaking in younger adults [35]. While thyroid nodules are more common and often multinodular in the elderly, our data suggest that, in this specific study population, advanced age is a high-risk feature rather than a reassuring one. This divergence might reflect a higher prevalence of aggressive tumor subtypes in the elderly subgroup in this cohort, consistent with the known association between older age and aggressive pathological features such as extrathyroidal extension and poorly differentiated histology [36,37]. Consequently, a “wait and see” approach for large nodules in older patients, often advocated to avoid overtreatment, may be less safe in this setting, warranting a lower threshold for biopsy.

Contrary to the well-established male predisposition to thyroid malignancy reported in global epidemiologic studies, our analysis found no significant association between male gender and malignancy (aOR 1.08; *p* = 0.844) [38]. This lack of male preponderance is an interesting deviation and may reflect regional epidemiological nuances. Studies have documented that thyroid cancer in the Gulf Cooperation Council (GCC) states exhibits one of the highest female-to-male ratios globally, potentially driven by hormonal, obesity-related, or reproductive factors specific to the region [39]. Additionally, the lack of association with multiplicity (solitary vs. multinodular) in this study cohort aligns with the updated consensus that malignancy risk is determined by the characteristics of the dominant nodule rather than the gland’s overall nodularity [40].

This study has several notable strengths. The use of strict inclusion and exclusion criteria and multivariable analysis adjusting for key confounders enhances the internal validity of the findings. In addition, the relatively large sample size and inclusion of a high-risk cohort from a tertiary referral center improve the clinical relevance and applicability of the results to surgical decision-making.

Several limitations should be considered when interpreting the findings of this study. The retrospective, single-center design introduces the possibility of selection bias, particularly as a tertiary-care population may overrepresent more complex or clinically suspicious thyroid nodules, potentially limiting generalizability to broader community settings. In addition, outcome classification based on a combination of histopathology, cytology, and clinical follow-up may introduce misclassification bias, particularly given the known false-negative rate of fine-needle aspiration in certain thyroid nodule subtypes.

Although multiple imputation (m = 20) was used to address missing ultrasound data and improve statistical efficiency, the validity of this approach relies on the untestable assumption that data were MAR, and violations of this assumption could introduce bias. Furthermore, while imputation allowed the use of the full cohort (n = 446), the effective events-per-variable ratio remained relatively low, which raises the possibility of model overfitting and instability of regression coefficients. A complete-case sensitivity analysis yielded broadly similar results, supporting the robustness of the primary findings.

Residual confounding cannot be excluded due to unmeasured variables such as iodine status, radiation exposure history, and interobserver variability in ultrasound interpretation. Additionally, practice patterns and diagnostic thresholds may have evolved over the extended study period (2018–2025), introducing potential temporal heterogeneity in assessment and management.

Lymph node status was available only for patients who underwent thyroidectomy (n = 126), among whom metastasis was present in 27 (21.4%), absent in 95 (75.4%), and undocumented in 4 (3.2%); as the majority of patients were managed non-surgically, systematic lymph node staging was not available for the full cohort, and restricting analysis to surgical patients would have introduced selection bias. Finally, the lack of stratification by histological subtype limits a more granular assessment of whether nodule size is differentially associated with specific thyroid cancer variants.

## 5. Conclusions

Thyroid nodule size is the strongest independent predictor of malignancy in this tertiary-care cohort, demonstrating a progressive increase in risk with larger nodules. The association between size and malignancy remained significant after adjustment for established clinical and sonographic risk factors, highlighting its central role in risk stratification. However, the variable performance of traditional ultrasound features in multivariable analysis, with select features such as hypoechogenicity and microcalcifications retaining independent predictive value, underscores the complexity of accurately predicting malignancy in thyroid nodules using morphology alone. These findings support the need to reconsider how nodule size is integrated into existing diagnostic frameworks. Future prospective multicenter studies are required to validate these observations, clarify histological subtype-specific associations, and refine size-based risk stratification models across diverse populations.

## Figures and Tables

**Table 1:** Summary of inclusion and exclusion criteria.

Category	Criteria
Inclusion Criteria	1. Age ≥ 18 years
	2. Ultrasonographically confirmed thyroid nodule with documented measurements
	3. Available thyroid function tests (thyroid-stimulating hormone [TSH], free thyroxine [T4])
	4. Fine-needle aspiration biopsy (FNAB) classified according to the Bethesda system **or** confirmed thyroidectomy with histopathological diagnosis
	5. Documented follow-up with confirmed malignancy status (histopathology or clinical consensus based on imaging)
Exclusion Criteria	1. Age < 18 years
	2. Prior thyroid cancer or other active malignancies
	3. Missing or incomplete thyroid nodule size measurements
	4. Unknown or uncertain malignancy status
	5. Inadequate follow-up documentation

**Table 2:** (**A**) Baseline demographic characteristics of the study population (n = 446). (**B**) Histopathological subtype distribution among malignant thyroid lesions (n = 91).

(**A**)			
**Variable**	**Benign (n = 355)**	**Malignant (n = 91)**	***p*** **-Value**
Age (years), mean ± SD	45.2 ± 13.1	46.7 ± 14.0	0.340 †
BMI (kg/m^2^), mean ± SD	27.3 ± 5.0	28.2 ± 5.6	0.154 †
Female sex, n (%)	278 (78.3)	72 (79.1)	0.867 ‡
Multiple nodules, n (%)	212 (59.7)	49 (62.0)	0.705 ‡
Comorbidities present, n (%)	116 (32.7)	24 (26.4)	0.632 ‡
Family history, n (%)	34 (9.6)	5 (5.6)	0.235 ‡
(**B**)			
**Histological Subtype**		**n**	**%**
Papillary thyroid carcinoma (all variants)		79	86.80%
Follicular thyroid carcinoma		5	5.50%
Hürthle cell carcinoma		2	2.20%
Medullary thyroid carcinoma		1	1.10%
Poorly differentiated thyroid carcinoma		1	1.10%
Mixed/unclassified variant		3	3.30%
**Total**		**91**	**100%**

Note: Data are presented as mean ± standard deviation for continuous variables and frequency (percentage) for categorical variables. Percentages are calculated based on valid cases excluding missing data. † Independent-samples *t*-test. ‡ Pearson chi-square test. BMI = body mass index; SD = standard deviation.

**Table 3:** Univariate analysis of clinical and ultrasound features.

Variable	Benign	Malignant	OR (95% CI)	*p*-Value
TSH level (mIU/L), median [IQR]	2.24 [2.20]	1.22 [2.57]	—	<0.001 §
Free T4 level (pmol/L), median [IQR]	1.29 [0.59]	1.45 [0.72]	—	0.010 §
Nodule size (cm), median [IQR]	1.80 [1.13]	2.30 [1.80]	—	0.015 §
Nodule size > 3 cm, n (%)	12.0%	28.6%	2.90 (1.71–4.93)	<0.001 ‡
Shape (taller-than-wide)	Wider-than-tall	Taller-than-wide	1.95 (0.88–4.35)	0.102 ‡
Composition (solid)	Mixed/Cystic	Solid	1.16 (0.66–2.02)	0.608 ‡

§ Mann–Whitney U test. ‡ Chi-square/logistic regression. TSH = thyroid-stimulating hormone; IQR = interquartile range; OR = odds ratio; CI = confidence interval.

**Table 4:** Multivariable logistic regression analysis for predictors of malignancy.

Predictor	aOR	95% CI	*p*-Value
Nodule Size (per 1 cm)	1.507	1.216–1.867	<0.001
Age	1.008	0.987–1.029	0.461
Gender (Male)	0.745	0.394–1.412	0.367
TSH Level	0.892	0.758–1.049	0.167
Free T4 Level	1.695	0.985–2.916	0.057
Nodule Multiplicity	0.939	0.523–1.667	0.829
Microcalcifications	1.857	1.009–3.415	0.047
Hypoechogenicity	2.070	1.120–3.826	0.020
Irregular Margins	1.576	0.803–3.091	0.186
Composition	1.002	0.541–1.856	0.996
Vascularity	1.780	0.993–3.192	0.053
Taller-than-Wide	2.324	0.957–5.642	0.062

Note: The model was adjusted for all variables listed above. aOR = adjusted odds ratio; CI = confidence interval; TSH = thyroid-stimulating hormone; T4 = thyroxine.

## Data Availability

The data presented in this study are available upon reasonable request from the corresponding author. The data are not publicly available due to privacy and ethical restrictions related to patient confidentiality.

## Abbreviations

The following abbreviations are used in this manuscript:

ATA	American Thyroid Association
ACR	American College of Radiology
TI-RADS	Thyroid Imaging Reporting and Data System
FNAB	Fine-Needle Aspiration Biopsy
TSH	Thyroid-Stimulating Hormone
BMI	Body Mass Index
OR	Odds Ratio
aOR	Adjusted Odds Ratio
CI	Confidence Interval
SD	Standard Deviation
PTC	Papillary Thyroid Carcinoma
AUS/FLUS	Atypia of Undetermined Significance/Follicular Lesion of Undetermined Significance
SPSS	Statistical Package for the Social Sciences
GCC	Gulf Cooperation Council

## References

[1] Dean D.S., Gharib H. (2008). Epidemiology of thyroid nodules. Best. Pract. Res. Clin. Endocrinol. Metab..

[2] Guth S., Theune U., Aberle J., Galach A., Bamberger C.M. (2009). Very high prevalence of thyroid nodules detected by high frequency (13 MHz) ultrasound examination. Eur. J. Clin. Investig..

[3] Durante C., Costante G., Lucisano G., Bruno R., Meringolo D., Paciaroni A., Puxeddu E., Torlontano M., Tumino S., Attard M. (2015). The natural history of benign thyroid nodules. JAMA.

[4] Demetriou E., Fokou M., Frangos S., Papageorgis P., Economides P.A., Economides A. (2023). Thyroid nodules and obesity. Life.

[5] Biondi B. (2023). Subclinical hypothyroidism in patients with obesity and metabolic syndrome: A narrative review. Nutrients.

[6] Adamska A., Raczkowski A., Stachurska Z., Kondraciuk M., Krętowski A.J., Adamski M., Kowalska I., Kamiński K.A. (2022). Body composition and serum concentration of thyroid hormones in euthyroid men and women from general population. J. Clin. Med..

[7] Haugen B.R., Alexander E.K., Bible K.C., Doherty G.M., Mandel S.J., Nikiforov Y.E., Pacini F., Randolph G.W., Sawka A.M., Schlumberger M. (2016). 2015 American Thyroid Association Management Guidelines for Adult Patients with Thyroid Nodules and Differentiated Thyroid Cancer. Thyroid.

[8] American College of Radiology (ACR) (2018). ACR Thyroid Imaging Reporting and Data System (TI-RADS): White Paper of the ACR TI-RADS Committee. J. Am. Coll. Radiol..

[9] Megwalu U.C. (2017). Risk of malignancy in thyroid nodules 4 cm or larger. Endocrinol. Metab..

[10] Kang S., Kim E., Lee S., Kim J.K., Lee C.R., Kang S.W., Lee J., Jeong J.J., Nam K.H., Chung W.Y. (2023). Do large thyroid nodules (≥4 cm) without suspicious cytology need surgery?. Front. Endocrinol..

[11] Bomeli S.R., LeBeau S.O., Ferris R.L. (2010). Evaluation of a thyroid nodule. Otolaryngol. Clin. N. Am..

[12] Jha A.K., Sinha A.K. (2020). A study to evaluate the size of thyroid nodules as an indicator for malignancy. Acad. J. Surg..

[13] Meng C., Wang W., Zhang Y., Li X. (2021). The influence of nodule size on the aggressiveness of thyroid carcinoma varies with patient’s age. Gland. Surg..

[14] Al Hamdan N., Ravichandran K., Al Sayyad J., Al Lawati J., Khazal Z., Al Khateeb F., Abdulwahab A., Al Asfour A. (2009). Incidence of cancer in Gulf Cooperation Council countries, 1998–2001. East Mediterr. Health J..

[15] Alsayyad J., Hamadeh R. (2007). Cancer incidence among the Bahraini population: A five-year (1998–2002) experience. Ann. Saudi Med..

[16] Alawainati M., Radhi M., Shawqi Z., Al Sharakhat A. (2019). Ultrasonographic and Pathological Features of Surgically Excised Thyroid Nodules: A Cross-Sectional Study. J. Bahrain Med. Soc..

[17] Hammad A.Y., Noureldine S.I., Hu T., Ibrahim Y., Masoodi H.M., Kandil E. (2016). A meta-analysis examining the independent association between thyroid nodule size and malignancy. Gland. Surg..

[18] Hong M.J., Na D.G., Baek J.H., Sung J.Y., Kim J.H. (2018). Impact of nodule size on malignancy risk differs according to the ultrasonography pattern of thyroid nodules. Korean J. Radiol..

[19] Jinih M., Faisal F., Abdalla K., Majeed M., Achakzai A.A., Heffron C., McCarthy J., Redmond H.P. (2020). Association between thyroid nodule size and malignancy rate. Ann. R. Coll. Surg. Engl..

[20] Cotter A., Jinih M. (2025). Thyroid nodule size and risk of malignancy: A systematic review. Discov. Oncol..

[21] Meko J.B., Norton J.A. (1995). Large cystic/solid thyroid nodules: A potential false-negative fine-needle aspiration. Surgery.

[22] Hanahan D., Weinberg R.A. (2011). Hallmarks of cancer: The next generation. Cell.

[23] Carmeliet P., Jain R.K. (2000). Angiogenesis in cancer and other diseases. Nature.

[24] Swanton C. (2012). Intratumor heterogeneity: Evolution through space and time. Cancer Res..

[25] Bongiovanni M., Spitale A., Faquin W.C., Mazzucchelli L., Baloch Z.W. (2012). The Bethesda System for Reporting Thyroid Cytopathology: A meta-analysis. Acta Cytol..

[26] Mehanna R., Murphy M., McCarthy J., O’Leary G., Tuthill A., Murphy M.S., Sheahan P. (2013). False negatives in thyroid cytology: Impact of large nodule size and follicular variant of papillary carcinoma. Laryngoscope.

[27] Pinchot S.N., Al-Wagih H., Schaefer S., Sippel R., Chen H. (2009). Accuracy of fine-needle aspiration biopsy for predicting neoplasm or carcinoma in thyroid nodules ≥4 cm. Arch. Surg..

[28] Wharry L.I., McCoy K.L., Stang M.T., Armstrong M.J., LeBeau S.O., Tublin M.E., Sholosh B., Silbermann A., Ohori N.P., Nikiforov Y.E. (2014). Thyroid nodules (≥4 cm): Can ultrasound and cytology reliably exclude cancer?. World J. Surg..

[29] Remonti L.R., Kramer C.K., Leitao C.B., Pinto L.C., Gross J.L. (2015). Thyroid ultrasound features and risk of carcinoma: A systematic review and meta-analysis of observational studies. Thyroid.

[30] Delfim R.L., Veiga L.C., Vidal A.P., Lopes F.P., Vaisman M., Teixeira P.D. (2017). Likelihood of malignancy in thyroid nodules according to a proposed Thyroid Imaging Reporting and Data System (TI-RADS) classification merging suspicious and benign ultrasound features. Arch. Endocrinol. Metab..

[31] Ha E.J., Na D.G., Moon W.J., Lee Y.H., Choi N. (2019). Diagnostic performance of ultrasound-based risk stratification systems for thyroid nodules: A multicenter study. Radiology.

[32] Middleton W.D., Teefey S.A., Reading C.C., Langer J.E., Beland M.D., Szabunio M.M., Desser T.S. (2017). Multi-institutional analysis of thyroid nodule risk stratification using ACR TI-RADS. AJR Am. J. Roentgenol..

[33] Grani G., Lamartina L., Ascoli V., Bosco D., Biffoni M., Giacomelli L., Maranghi M., Falcone R., Ramundo V., Cantisani V. (2019). Reducing the number of unnecessary thyroid biopsies while improving diagnostic accuracy. J. Clin. Endocrinol. Metab..

[34] Sutton W., Canner J.K., Rooper L.M., Prescott J.D., Zeiger M.A., Mathur A. (2021). Is patient age associated with risk of malignancy in a ≥4 cm cytologically benign thyroid nodule?. Am. J. Surg..

[35] Kwong N., Medici M., Angell T.E., Liu X., Marqusee E., Cibas E.S., Krane J.F., Barletta J.A., Kim M.I., Larsen P.R. (2015). The Influence of Patient Age on Thyroid Nodule Malignancy Risk Requiring Extended Monitoring. J. Clin. Endocrinol. Metab..

[36] Shah S., Boucai L. (2018). Effect of Age on Response to Therapy and Mortality in Patients with Thyroid Cancer at High Risk of Recurrence. J. Clin. Endocrinol. Metab..

[37] Johar J., Britton H., Wiseman S.M. (2020). Older patients with differentiated thyroid cancer exhibit more aggressive pathological characteristics than younger patients. Can. J. Surg..

[38] Rahbari R., Zhang L., Kebebew E. (2010). Thyroid cancer gender disparity. Future Oncol..

[39] Al-Zahrani A.S., Ravichandran K., Al-Hamdan N. (2002). The epidemiology of thyroid cancer in the Gulf Cooperation Council states. J. Fam. Community Med..

[40] Frates M.C., Benson C.B., Doubilet P.M., Kunreuther E., Contreras M., Cibas E.S., Orcutt J., Moore F.D., Larsen P.R., Marqusee E. (2006). Prevalence and distribution of carcinoma in patients with solitary and multiple thyroid nodules on sonography. J. Clin. Endocrinol. Metab..

